# Clinical and school-based intervention strategies for youth obesity prevention: A systematic review

**DOI:** 10.3389/fspor.2022.906857

**Published:** 2023-02-27

**Authors:** Théo Caron, Paquito Bernard, Tegwen Gadais

**Affiliations:** ^1^ Faculté des Sports et de l’EP, Université D’Artois, Liévin, France; ^2^ Département des Sciences de l’activité physique, Université du Québec à Montréal, Montréal, QC, Canada; ^3^ Research Center, University Institute of Mental Health at Montreal, Montréal, QC, Canada; ^4^ UNESCO Chair in Curriculum Development (UCCD), Université du Québec à Montréal, Montréal, QC, Canada

**Keywords:** obesity, youth, clinical, school, intervention strategies, prevention

## Abstract

**Introduction:**

In the last couple of decades, numerous intervention strategies (ISs) have been formulated in school/community or clinical sectors using physical activity (PA) in order to prevent youth obesity because they have been highly effective in addressing this issue. These two sectors have revealed some interesting information in terms of efficient results and best practice mechanisms, but comparisons between them to learn about their functioning have been rare.

**Methods:**

Therefore, the aim of this systematic review was to analyze and synthesize PA ISs from school/community or clinical domains for the period 2013-2017, in French or English, targeting youths aged 5-19 years old through primary, secondary, and tertiary prevention.

**Results:**

In total, 68 full articles were reserved for data extraction and synthesis and 617 were excluded because they did not meet eligibility criteria (61 of 68 were kept for the final analysis). The results identified a number of differences between the studies of the various IS sectors and also a third type of IS, mixed sector. Mixed ISs (clinical and school-community) have a special advantage because they can benefit from the strengths of both school/community-based and clinical-based ISs. Mixed ISs showed the most promising results. This review also highlighted the differences between sectors and their ISs in terms of intervention teams, prevention objectives, duration, materials, and efficiency.

**Conclusion:**

Future studies should focus on establishing a prevention program in a given geographical area involving all stakeholders with their respective skills/knowledge, in the area of decision-making and in the development of ISs, to ensure that the program is the most efficient and best adapted to its environment.

## 1. Introduction

Nowadays, it is well known that obesity is a chronic pathology with multifactorial origins, defined by the World Health Organization (WHO) as an abnormal or excessive fat accumulation that presents a risk to health (e.g., vascular, endocrine) (1, 2). A study reported that in the last four decades, the number of young obese children (aged 5–19) multiplied by 10 worldwide (3). Another study reported that the rate of youth obesity prevalence increased by 47% in the last three decades (4). To help control this disease condition, the WHO made some recommendations [e.g., physical activity (PA); nutrition] (5, 6) to be followed, in order to adopt a healthy lifestyle and reduce health problems and the risk of obesity.

In parallel, it is also known that PA has many health benefits (physical, mental, and social, among others) that can help reduce obesity and maintain a certain weight when combined with nutrition (7). Moreover, the adoption of a healthy lifestyle during childhood or adolescence tends to spill over into adulthood. Nevertheless, 80% of youths aged 13–15 do not follow those PA recommendations (8) and tend to lead a sedentary lifestyle (9). This sedentariness and inactivity could lead to overweight or obesity (10). To address this issue and curb this tendency, intervention strategies (ISs) and programs integrating PA have been developed and implemented in different settings to prevent overweight and obesity (11). According to Gadais (11), ISs are initiatives and programs with thematic content and events that directly or indirectly aim to facilitate people to adopt healthy lifestyles for the benefit of their immediate and future health. When we look closely at the literature on obesity prevention (12), a few different settings emerge, and two of them have been widely studied (13, 14): the clinical setting on how to manage childhood obesity and the school/community setting on how to deal with obesity prevention and the role of the school in such prevention. It is from this perspective that we decided to focus our work on these two promising intervention sectors.

As we suggested earlier and to quote Lydecker et al. (15), “prevention assumes that individuals have some degree of susceptibility to obesity and would benefit from medical and psychosocial interventions to counter that susceptibility” (15). If the degree of susceptibility to obesity varies from one individual to another, prevention must also take place at different levels: primary, secondary, and tertiary. Primary prevention targets every individual without any distinction, for example, advertisements on television that invite people to be active and eat better. Secondary prevention targets subsamples of the population: people at risk of becoming overweight or obese, for example, children in the upper BMI range or who engage in very little physical activity. Tertiary prevention targets specific individuals who are already overweight or obese with complications, in this case, interventions that aim to help people obtain sufficient weight loss to reduce comorbidities (15–17). These three types of interventions are generally implemented in two major sectors: clinical or school and community sectors.

A clinical setting is a place where people are treated (e.g., hospital, health center). In this context, clinical ISs seem to contain better financial resources (e.g., exergaming) (18) and human resources/expertise to act as a source of quality and reliable information (19). Clinical ISs have also shown good results in the fight against childhood obesity (20–22), making it an important contributor in the management of obesity. Nevertheless, not all studies show good results, as prevention does not involve only treatment, which is mostly the last step of prevention, thus making the ISs of the other sector also useful.

The school and community sector can be seen as a place dedicated to learning where children develop their knowledge and skills (e.g., physical, social, cognitive skills). Many authors agree that school is a privileged place for the prevention of obesity (23–25). Indeed, children and adolescents spend most of their time at school and it is “possible to globally reach the population of interest without stigmatizing or discriminating and without being primarily dependent on families” (26). According to these authors, the school/community sector could assume an important role to promote positive change in children's lifestyles, in order to make them adopt a healthy way of life (11, 27). Yet, some studies have demonstrated that school-based obesity prevention interventions with children have produced limited efficacy (28, 29), generally lacking in financial or human resources, among others.

According to the literature, school/community-based ISs and clinical ISs seem to be different because they do not employ the intervention on the same level of childhood obesity prevention. Interestingly, both seem to show promising results in preventing obesity. Therefore, a question arises: Could it be possible to consider a global prevention strategy (primary, secondary, and tertiary levels) to reduce youth obesity prevalence and incidence in the coming years by integrating the best practices from one sector into another? We, therefore, sought to know if there were relevant elements in the ISs from these two sectors that would help formulate effective strategies for the prevention of obesity among young people through mutual enrichment.

The aims of this study were to

(1) prepare an extensive inventory on the recent literature regarding programs and ISs that aimed at preventing youth obesity, from clinical or school-community perspectives;
(2) extract information in order to identify the mechanisms that make programs effective in a clinical or school/community sector;
(3) propose some recommendations from the point of view of both sectors (clinical and school/community) and improve the current ISs for future studies.

## 2. Methods

**Figure 1:**
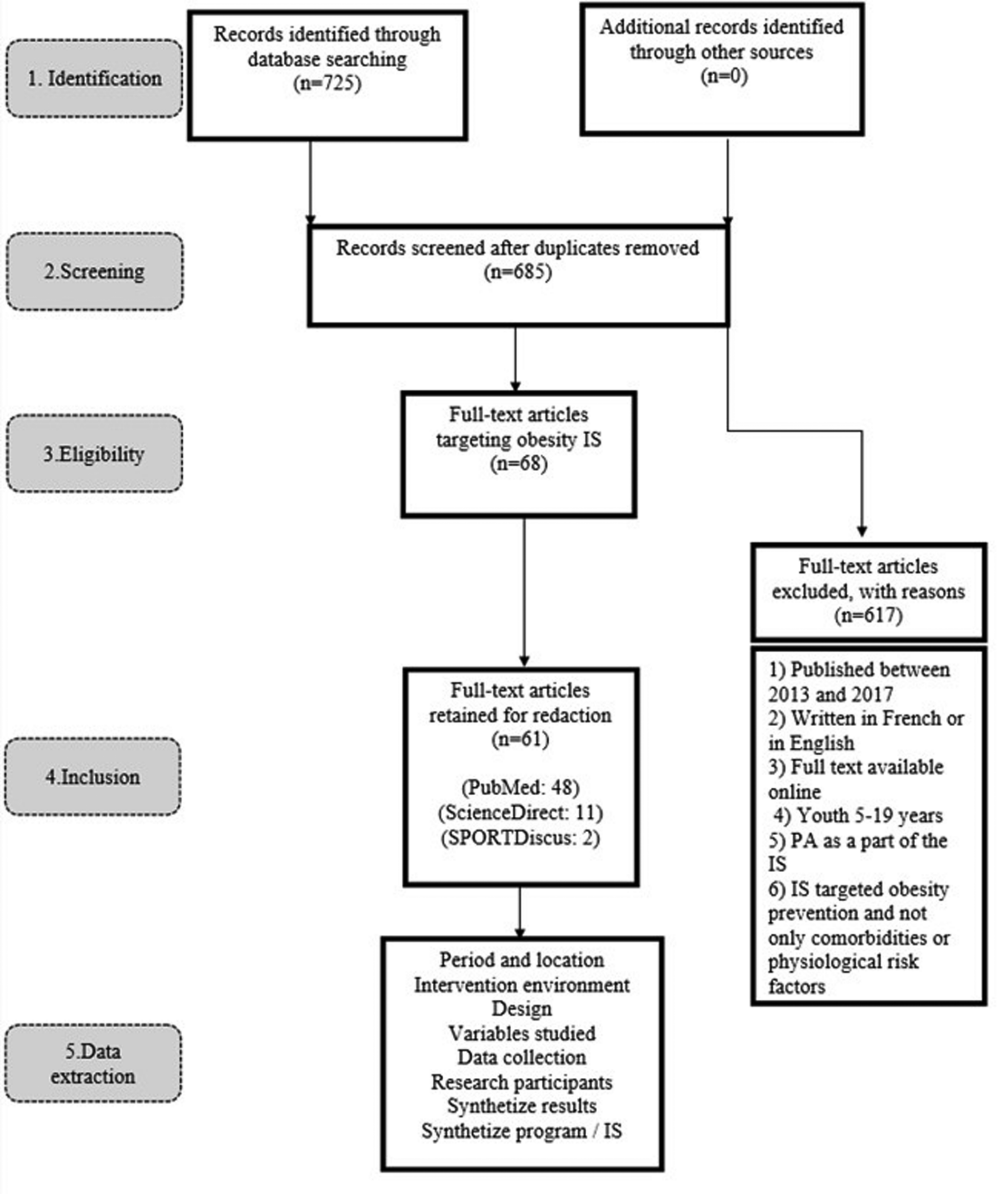
PRISMA flow chart.

To conduct this systematic review, we followed the six steps of the PRISMA (30) for preparing a flow chart (Figure 1).

### 2.1. Phase 1: Identification of studies

In the first phase, an exhaustive search through computerized databases was performed to identify scientific publications targeting youth obesity prevention. Particularly, we used specific search equations to conduct the first overall research. Relevant articles were identified by means of a computerized search through three databases (i.e., SPORTDiscus, PubMed, and ScienceDirect) with different combinations of keywords (e.g., program; obese; physical activity, child) (Appendix A) and Equations/Meshterms (Appendix B).

### 2.2. Phase 2: Screening

In phase two, 40 duplicates were eliminated and 685 papers were identified. We started looking for non-systematic reviews and prioritized peer-reviewed articles. Some reports from credible organizations such as government agencies, international agencies, or academic centers were also included in our research (e.g., WHO), but none of them were finally considered.

### 2.3. Phase 3: Eligibility

The selection of the abstract was done on the basis of inclusion criteria. To dispel any doubts on this process, the third author was consulted. Six inclusion criteria were applied for article eligibility: (1) articles published between 2013 and 2017, (2) those written in French or in English, (3) full text available online, (4) youths 5–19 years’ old targeted by studies, (5) PA as a part of ISs, (6) ISs that targeted obesity prevention and not only comorbidities or physiological risk factors (e.g., diabetes). In total, 68 full articles were reserved for data extraction and synthesis and 617 were excluded because they did not meet the eligibility criteria.

### 2.4. Phase 4: Inclusion

The fourth phase of the literature search involved obtaining copies of the articles previously identified in phase 3. After collecting the articles, the overall sample was reassessed. A complete reading was made in order to eliminate non-relevant articles (according to inclusion criteria). Finally, 61 articles were retained for data extraction by meeting all inclusion criteria and focusing on the topic of this study.

### 2.5. Phase 5: Data extraction

A data sheet was used to extract information concerning the date of the study; intervention sector (school/community or clinical); design (collaborative ISs or not); variables studied; data collection (equipment, hardware); participants (non-obese, overweight, obese, age); synthesized results; synthesized ISs. The first and the second author performed data extraction. To dispel any doubts on this process, data synthesis tables were discussed until agreement was reached on the presentation form and what should be extracted. The completion of phase 5 marked the start of the content analysis of the 61 articles.

### 2.6. Phase 6: Data analysis

To identify the differences, the results will be presented in five different categories (i.e., intervention team, prevention objective, duration, material, and efficiency). As previously mentioned, the intervention sectors (clinical and school/community) are different from each other but show interesting results (10–12). Therefore, it is interesting to see who conducted the intervention, its purpose, the means available, how long the ISs lasted, and the results.

The first author carried out the initial selection in the literature search on the basis of the abstract and title (*n* = 725). He also performed the initial analysis of the data and wrote the first draft of the manuscript. The second and third authors helped with data analysis and result presentation. They also revised the entire manuscript.

## 3. Results

### 3.1. School and community ISs

#### 3.1.1. Intervention team

Of the 36 school/community-based studies (Table 1), 11 used one type of stakeholder. Of these 11, 6 studies involved only teachers (23, 31–35). For example, Grao-Cruces et al. (32) mentioned “weekly follow-up and control by PE teachers” and Sacchetti et al. (23) “taught by the ordinary classroom teacher.” The use of the teacher as the only intervener increases from 54.5% (6/11) to 75% (6/8) (36–38) if we exclude studies occurring only in the community. Twenty-five studies used at least two types of stakeholders (18, 24, 25, 39–60), and eight involved health professionals (e.g., doctors, nurses, psychiatrists) (18, 25, 39, 42, 45, 48, 59, 60). Among these eight, two relied solely on school nurses (42, 48). Globally, six studies involved non-school-based stakeholders with experience in healthcare.

**Table 1:** Illustrations of studies for school/community ISs.

Authors, year	Participants, prevention type	Material and variables	Program/ISs summary	Results summary	City (country)
Safdie et al. (2013)	RCT, youth aged 9–11 years old (*n*: 886) Primary/secondary prevention (impact on obesity risk factors) Collaboration between the physical education general direction, The National Institute of Public Health, The Federal Administration of Educational Services	Food and beverage availability, food intake PA levels and quality of PE lessons BMI-SD	The basic program focused on nutrition, physical activity, communication, and education. The Plus program focused on nutrition, physical education, communication, and education but with workshops and not only booklets	- The availability of recommended foods increased significantly (*p* < 0.05) - (*p* = 0.06) children in basic schools and (=0.03) in Plus schools maintained a status of reaching cut-off for steps in school relative to students in the control group - The intervention had no significant effect on the prevalence of overweight and obesity or children's BMI	Mexico City (Mexico)
Wright et al. (2013).	RCT, youth aged 8–12 years (*n*: 251) Secondary prevention (BMI > 85th percentile) Community collaboration and University partnership with school	BMI BMI z-score Health behaviors knowledge	Phase 1 (KNF© intervention): Focused on youth PA (practice) and nutrition (group sessions with parents) Parental involvement (sessions on obesity consequences and healthy lifestyle) Phase 2 (“Environmental” activities): Healthcare offered to participating children Establishment of a school health advisory council (establishment of health policy, providing newsletter to parents, offering seminars to school staff and parents on health promotion)	- Significant decrease in BMI z-for girls (*p* < 0.005) stays at 4 and 12 months. - Boys and girls fit with the recommendations of 60 min of daily PA (*p* = 0.002 and *p* = 0.005) at 12 months. - Boys and girls increased their attendance in PE classes (*p* = 0.003 and *p* = 0.002) - Boys and girls decreased their TV use at 4 months but maintained only by boys at 12 months (*p* = 0.03)	Los Angeles (USA)
Staiano et al. (2013)	RCT, youth aged 15–19 years (*n*: 54) Secondary/tertiary prevention (BMI percentile >75th)	Anthropometric BMI z-score Psychosocial variables (e.g., Rosenberg self-esteem scale)	Competitive exergaming 30–60 min of exergaming per school day during 7 months—1 coordinator → encourage daily exergaming + ensure the maintenance of the social environment of the classes Goal = Earn more points than opponents Cooperative exergaming: 30–60 min of exergaming per school day during 7 months - 1 coordinator → encourage daily exergaming + ensure the maintenance of the social environment of the classes Goal = Earn the most points with partner Control: Usual activities	- Cooperative group lost significantly more weight than the control group (*p* = 0.021) - Competitive group did not experience significant weight variation, compared with others	Georgetown (USA)

ISs, intervention strategies; PA, physical activity RCT, randomized controlled trial ; PE, physical education.

#### 3.1.2. Prevention objective

Of all the studies aimed at preventing childhood obesity through school/community ISs, nine dealt with secondary or tertiary obesity prevention, since they were only interested in young people who were already overweight or obese (18, 25, 37, 38, 47, 48, 51, 59, 60). For example, Larsen et al. (48) selected “One hundred fifteen 11–13 year-old children with overweight and obesity” and Wright et al. (25) mentioned, “Students (*n* = 251) were English or Spanish speaking, had a BMI > the 85th percentile”. The majority of the studies (23, 24, 27, 31–36, 39–46, 49, 50, 52–58, 61, 62) directed their attention toward a relatively primary prevention of obesity and did not necessarily target an overweight or obese population but rather an entire population. For example, Lau et al. (49) mentioned, “the average BMI was 17, which was within a healthy range.” These studies tended to include children “at risk” as their participants because of the absence of selection criteria. For example, Smith et al.'s (57) intervention group included 2 underweight children, 110 normal weight, 39 overweight, and 30 obese.

#### 3.1.3. Duration

The duration of 19 of the studies was a year or more (23, 24, 31, 33, 36, 39, 41–43, 45, 51, 52, 54–56, 58, 59, 61, 62). For example, Erfle and Gamble (42) reported “30 min of daily PE throughout 1 academic year” and Santos et al. (24) mentioned, “performed during the 2009–2010 school year.” Of the 17 remaining with a shorter duration, 14 of them lasted 6 months or less (18, 25, 32, 34, 37, 38, 40, 44, 46–49, 53, 60). Larsen et al. (48) used “the six-week intervention” and Parra-Medina et al. (53) “a 12-week family focused healthy lifestyle program.”

#### 3.1.4. Material

Studies from the school/community sector did not automatically consider BMI with age and sex in their anthropometric measurement (e.g., BMI *z*-score, BMI percentile). Two studies did not consider anthropometric measurement in their outcomes (40, 46) and two others considered only BMI (not for age and sex) (49, 59). Of the 34 studies with anthropometric measurements, 23 were based on a measurement related to BMI or abdominal circumferences (e.g., BMI score, waist circumference). Other studies (indicating anthropometric measurements) systematically considered at least a second objective measure such as dual energy x-ray absorptiometry (DXA) (one study), skinfold thickness (three studies), or impedance (seven studies). Ning et al. (37) mentioned that “body composition was assessed by bioelectrical impedance analysis (…) body composition was also estimated at baseline and 6 months using dual x-ray absorptiometry” and Johnston et al. (47) mentioned that “body composition was assessed using triceps skinfold thickness.” For studies that assessed PA measurement (32/36), 20 used one objective measurement (not a self-reported). Among these, the most common instruments were accelerometer (9 studies), pedometer (5 studies), and various fitness tests (13 studies) (e.g., Fitnessgram). For example, Larsen et al. (48) used a “progressive bicycle ergometer protocol (…) Actigraph GT3X + for ten consecutive days” and Grao-Cruces et al. (32) reported that “a pedometer was used for evaluation and follow-up purposes.” Finally, regarding nutrition, 19 studies collected data. Most of them were carried out through three types of instruments: survey (15 studies), recall/diary (6 studies), and interview (1 study). For example, Ning et al. (37) mentioned that it was “assessed by a 48-hr diet recall.”

#### 3.1.5. Efficacy

In the school/community sector, the major objective tended to be the prevention of obesity, and interestingly, “lose weight” was not the first goal.

However, 26 studies still presented significant positive results regarding the anthropometric measurements of the participants (e.g., BMI, waist circumferences) (18, 23–25, 31–35, 37–39, 41, 42, 45, 47, 48, 50–52, 55, 56, 59–62). Nevertheless, some studies showed effects only on a part of the population (25, 33) or “mixed effects” (23, 41, 56). Of the 10 remaining studies, which did not clearly show an effect on BMI, 9 had, at least, a significant influence on health factors (36, 40, 43, 44, 46, 49, 54, 57, 58) (e.g., physical, psychological, or nutritional). To illustrate, Smith et al. (57) showed that “significant intervention effects were found for screen time (mean SE: −30 ± 10.08 min/day; *p* = 0.03), sugar-sweetened beverage consumption (mean: −0.6 ± 0.26 glass/day; *p* = 0.01).” Only the article by Parra-Medina et al. (53) showed no significantly interesting effect on children because “child participants that completed the program (*n* = 72) showed no improvements.”

### 3.2. Clinical ISs

#### 3.2.1. Intervention team

Of the 19 studies we identified (Table 2) in the literature and that were carried out in a clinical setting, 3 (19, 63, 64) used a single type of contributor. It was systematically a doctor who delivered recommendations on psychological, nutritional, or PA. For Brennan et al. (63), “the clinician discussed topics such as physical activity, nutrition, helpful thoughts and emotions,” and for Davis et al. (64), “the clinician covered several topics such as self-esteem, energy balance, portion size, screen time and sedentary.” The 16 remaining studies included a multidisciplinary team composed of at least two specialists (65–80). For example, for Nemet et al. (73), “the intervention team was composed of 3 specialists: dietitian, coach and physician” and Endevelt et al. (68) used a “multidisciplinary team including a pediatrician, a dietician, a physical activity expert, and a social worker.”

**Table 2:** Illustrations of studies for clinical ISs.

Authors, year	Participants, prevention type	Material and variables	Program/ISs summary	Results summary	City (country)
Martín-García et al. (2017)	Youth aged 7–16 years old (*n*: 61) Secondary/tertiary prevention (BMI > 85th percentile)	Anthropometric (height, weight, BMI *z*-score) Body composition (DXA) Eating behaviors PA intensity and enjoyment Health-related quality of life	Focused on recreational PA games (mainly aerobic games, at least 10 min per game) at a high intensity; 75.5% of child's maximal HR (mean 151 ± 13 bpm)	- Significant decrease in whole-body fat mass and % body fat mass for boys (*p* < 0.05 and *p* < 0.001, respectively) - Significant increase in (*p* = 0.003) lean mass (whole body) - VPA reduce overeating behaviors especially linked to negative mood state (reduction of emotional eating traits)	Madrid (Spain)
Serra-Paya et al. (2015)	Youth (B/G) 6–12 years old (*n*: 113) Secondary/tertiary prevention (BMI > 85th percentile)	Anthropometric (height, weight, BMI *z*-score) Dietary habits PA and sedentary time	Supervised PA for the child (3 × 1 h/week) Practical and theoretical Sessions for parents (1 × 1 h/week) Weekends of activities offered outside the family (3×) Session on good behaviors to adopt	Decrease BMI: - If attendance ratio = 0.547 (*p* < 0.001) - Improve LPA and MVPA (*p* < 0.001) - Improve MVPA by 2.5 h/day - Increase MVPA in all analysis subgroups (puberty vs. not; boys vs. girls) - Increase fruit consumption (3/day and decrease in sugar-sweetened juices/soft drinks)	Leida (Spain)
Staiano et al. (2017)	Young girls, 14–18 years old (*n*: 41) Secondary/tertiary prevention (BMI > 85th percentile)	Anthropometric (height, weight, BMI *z*-score) Body composition (DXA) Cardiovascular risk factors (blood sample, blood pressure, resting electrocardiogram)	Supervised (“gaming coaches”) dance exergaming sessions with a self-selected intensity, dance partner, game. (60 min, 3×x per week for 12 weeks)	- Per protocol analysis (attendance >75%): significant improvement in BMD for trunk and spine (*p* = 0.03 and *p* = 0.008, respectively) - Per protocol analysis (steps per session >2,600): significant decrease in leg fat % (*p* = 0.049), subcutaneous adipose tissue (*p* = 0.02) and total adipose tissue (*p* = 0.03)	Baton-Rouge (USA)
Marild et al. (2013)	Youths 9–13 years old (*n*: 64) Tertiary prevention (BMI-SD > IOTF-30)	Anthropometric (height, weight, BMI *z*-score, WHtR) Cardiovascular risk factors (blood sample) Pubertal stage (Tanner stages)	NDT: 8 × 1 h nurse visits for 1 year (monitor weight development, reinforce diet messages and try to reduce inactivity) 4 × 1 h dietician visits for 1 year (dietary habits) NDPT: 4 × 1 h nurse visits for 1 year (monitor weight development and reinforce diet messages) 4 × 1 h dietician visits for 1 year (dietary habits) 4 × 1 h physiotherapist visits for1 year (reduce inactivity, change transportation, use pedometer for motivation and diary to register steps, reduce inactivity, stimulate child to participate in PE lessons at school, and talk about PA recommendations)	- No significant differences were observed between NDPT and NDT interventions. - Significant decrease in BMI for NDPT and NDT (*p* = 0.0007 and *p* = 0.002 respectively) compared with the non-intervention group - Significant decrease in BMI-SD for NDPT and NDT (*p* = 0.0005 and *p* = 0.002 respectively) compared with the non-intervention group	Alingsås, Göteborg, Trollhättan, and Skövde (Sweden)

ISs, intervention strategies; PA, physical activity; DXA, dual energy x-ray absorptiometry; BMD, bone mass density; NDT, Nurse-Dietician management treatment; NDPT, Nurse-Dietician-physiotherapist management treatment. HR, heart rate; VPA, vigourous physical activity; LPA, low physical activity; MVPA, moderate to vigourous physical activity.

#### 3.2.2. Prevention objective

None of the studies targeted the primary prevention of obesity and 18 of them worked on secondary or tertiary prevention of obesity, because they only targeted participants with a BMI >85th percentile. For example, Staiano et al. (79) selected only participants with a BMI percentile >85th, according to the Center of Disease Control (CDC) growth chart; and Serra-Paya et al. (76) selected children overweight or obese, according to the International Obesity Task Force (IOTF) criteria. Only one study (19) used a BMI between the 75th and the 95th percentiles as an inclusion criterion. Nevertheless, participants were judged at risk of weight gain due to their BMI, based on their last medical consultation.

#### 3.2.3. Duration

For the duration of the ISs, 16 studies covered at least 1 year (63–69, 71–79). Luca et al. (70) mentioned a “2-year interdisciplinary obesity management program.” Moreover, 10/19 studies had an effective duration of less than or equal to 6 months (64, 65, 69, 72–75, 77–79). Martín-García et al. (72) implemented a 3-month vigorous physical activity plan and Staiano et al. (78) a 12-week group exergaming intervention. It should also be noted that all studies covering one or more years consisted of only a few meetings throughout the year. To illustrate, for Stettler et al. (19), the ISs consisted of 12 meetings of 15–25 min over 12 months, and for Luca et al. (70), it was 6 meetings of 2 h per week, then 1.5 h every 2 weeks the first year, and 1.5 h monthly until the 18th month.

#### 3.2.4. Material

Anthropometric measurement at the clinical level consistently considered BMI by age and sex (BMI score). Nevertheless, in 10 studies, BMI was coupled with a second measure related to body composition and something more (19, 63, 65, 69, 71, 72, 76–79) [DXA (4 studies), skinfold thickness (2 studies), Waist to Height Ratio (WHtR) (2 studies), and impedance (1 study)]. For example, Staiano et al. (78) used DXA to assess body composition and quantify body fat, and Gerards et al. (69) measured skinfold thickness to evaluate the percentage of body fat. With regard to PA, 12 studies used one or more objective measurements (63, 65, 66, 69–74, 76, 78, 79). Of these 12, the pedometer was used in 2 studies; PA was tested in 5 studies, and accelerometers in 7 studies. Brennan et al. (63) used a cycle ergometer test to assess cardiovascular fitness and participants had to get an accelerometer fixed on them to have their physical activity assessed. With regard to nutrition, measurements were made in 14 studies (19, 63–70, 72–76) and three tools were frequently used: survey (3 studies), interview (4 studies), and dietary recall (5 studies). Nemet et al. (74) mentioned the “use of a 48-h dietary recall”; Davis et al. (64) spoke about the “use of a 24-h dietary recall,” and Brennan et al. (63) referred to the “use of a dietary checklist and of the Fat, Fruit and Vegetables Diet Questionnaire (FFVDQ).” It should be noted that some studies did not clearly mention their measurement instruments.

#### 3.2.5. Efficacy

In the context of secondary or tertiary prevention of obesity, one of the main objectives remained BMI decrease and fat loss in favor of lean mass. Out of 19, 14 (73.68%) studies showed significant effects on BMI or participant body fat (19, 63–65, 68, 71–75, 77–80). For example, Marild et al. (71) “reported a significant reduction in BMI and BMI-SD in the Nurse-Dietician-Physiotherapist managed treatment compared to the control group with obesity (*p* = 0.0007 and *p* = 0.0005 respectively).” Nevertheless, some studies showed significant effects only on some of their participants. For example, Martín-García et al. (72) “found that, in the intervention group, boys decreased their whole-body fat mass (*p* < 0.04) and reduced their percentage of body fat (*p* < 0.001); moreover, boys’ body lean mass increased significantly (*p* = 0.003).” Of the five other studies that did not have a direct effect on BMI, four had at least a significant influence on physical, psychological, or nutritional health factors (66, 69, 70, 76). Furthermore, in these studies, the intervention group was compared with a control group performing a “less advanced” intervention. Serra-Paya et al. (76) mentioned that “the intervention group received organized physical activity sessions, theoretical and practical sessions for parents, behavior counselling for children and parents, 3 weekend activities organized outside the family for children; unlike the counselling group that received only the behavior counselling sessions.” Only one article showed no effects (67).

### 3.3. Mixed ISs

#### 3.3.1. Intervention team

Of the six studies we identified (Table 3) and that were carried out in a “mixed” setting (school/community and clinical), all of them used at least two types of contributors, with one (or more) having health-related skills or knowledge. Rito et al. (81) reported that “four individual counselling sessions performed by trained nutritionists (…) healthy cooking workshops performed by a certified renowned ‘chef’ in a school kitchen.” All of these studies used a multidisciplinary team having in common a dietician. Maatoug et al. (82) mentioned that the ISs “included a multidisciplinary team with a pediatrician, dietician, physical activity teacher and psychologist.”

**Table 3:** Illustrations of studies for mixed ISs.

Authors, year	Participants, prevention type	Material and variables	Program/ISs summary	Results summary	City (country)
Morano et al. (2016)	Youth (B/G) 11.3 ± 0.4 years (*n* = 18) Secondary/tertiary prevention (BMI ≥ 85th percentile; CDC)	BMI-SD/percentile Anthropometric Physical fitness (Eurofit, Fitnessgram) Dietary habits PA enjoyment and perceived PA abilities; HRQoL	Exercise training and fun-type PA 2 × 2 h/week PA diary review 1 × 30 min/week in groups Nutrition counseling sessions 3 times during the 6-month intervention + 1 time at the beginning to give recommendations. Parents monitor their child on the completion of the PA diary	- Significant decrease in BMI variables (e.g., BMI *z*-score *p* = 0.001; BMI percentile *p* = 0.001) and % body fat (*p* < 0.001) - Skinfold thickness reduction (e.g., biceps, *p* < 0.001; Subscapular *p* = 0.008) except for triceps skinfold (*p* = 0.363) - Physical performance significantly improved (e.g., 10 m sprint, *p* < 0.001) as Perceived PA (*p* = 0.026) and Enjoyment of PA (*p* = 0.035) - Psychosocial health improved significantly (*p* = 0.048) but there was no significant effect on physical health. - Better dietary habits showed [e.g., reduction in caloric intake (kcal/day) *p* < 0.001]	Parisi-De-Sanctis, Foggia (Italy)
Maatoug et al. (2015)	Youth (B/G) 13.1 ± 0.96 years (*n*: 317) Secondary/tertiary prevention (BMI-SD >1; WHO) Transfer of a clinical strategy to a school-based intervention	BMI-SD Anthropometric PA expenditure Dietary intake	2 arms: collective intervention: PA group sessions on - healthy eating - self-esteem - snacking PA sessions proposed by teachers Individual intervention: Only obese meeting on - self-esteem and depression screening - dietician consultation - causes of obesity - educate and motivate participants on healthy eating and PA habits	- BMI-SD decrease pre-post (*p* < 0.001) and after follow-up 4 months (*p* < 0.001) - BMI-SD decrease pre-post (*p* < 0.001) and after follow-up 4 months (*p* = 0.230) in the control group - Decrease in caloric intake (*p* < 0.001) pre-post in CG and IG - No PA drop in IG (*p* = 0.690) contrary to CG (*p* = 0.001)	Sousse (Tunisia)
Rito et al. (2013)	Youth (B/G) 6–10 years (*n*: 266) Secondary/tertiary prevention (BMI ≥ 85th percentile; CDC)	BMI percentile Anthropometric PA and sedentary Dietary intake Nutritional and physical activity knowledge, attitudes and behavior	Health center (individual) 4 × 1 h nutrition counseling sessions Family “healthy cooking” workshop 1 × 3 h cooking practice for skills development and knowledge (e.g., food preservation and storage) + POZ recipe book School intervention Child: 6 h of intervention focusing on healthy eating and PA Parents: 3 h of intervention focusing on healthy eating and PA (improve knowledge + support their child) + Brochures Teachers “Nutrition and physical activity sheets” given to facilitate additional initiatives in classrooms	- Significant decrease in BMI variables (e.g., BMI percentile, CI 95% −2.2; −1.3; *p* < 0.001) - No significant effect on dietary variables, except for fiber consumption (*p* = 0.005) - VPA improvement (CI 95% 0.1; 0.5; *p* = 0.008) Nutrition knowledge and attitude improvement (Knowledge, CI 95% 4.6; 7.1; *p* < 0.001 and attitude CI 95% 0.9; 1.6; *p* < 0.001)	Melgaço, Cascais, Mealhada, Beja, and Silves (Portugal)

ISs, intervention strategies; CDC, Center of Disease Control; PA, physical activity; WHO, World Health Organization. CG, control group; IG, intervention group; B/G, blood glucose; HRQoL, health-related quality of life; POZ, POZ scale https://www.pozqol.org/about-pozqol/; VPA, vigorous physical activity

#### 3.3.2. Prevention objective

All these studies were part of a secondary or a tertiary prevention of obesity, because they were interested only in children who were already overweight or obese. Four of them were based on data on overweight and obesity for age and sex of CDC (BMI > 85th percentile). For example, Morano et al. (83) selected participants “with a BMI ≥ 85th percentile for age and sex according to the CDC growth reference” and Sanders et al. (84) selected “overweight or obese based on the CDC growth chart.” The two remaining studies were based on different data from the WHO: in Maatoug et al. (82), “*Z*-score were derived using the world health organization references” or from English references; in Kokkvoll et al. (85), “≥98th percentile according to the UK references.” None of these studies were directly concerned with the primary prevention of the pathology.

#### 3.3.3. Duration

With regard to these mixed studies, two had a duration above or equal to 1 year (82, 85). Kokkvol et al.'s (85) ISs lasted 2 years, and Maatoug et al.'s (82) ISs (50%) lasted 1 year and the remaining (50%) lasted 6 months or less (81, 83, 84). Sanders et al. (84) formulated a 4-week IS. Only Rieder et al.’s (86) strategies had an “intermediate” duration equal to 9 months. It should be noted that, unlike the clinical studies previously seen, the frequency of proposed activities or meetings with professionals was higher in mixed programs lasting 1 year or more. For example, Kokkvoll et al. (85) used “weekly group-based physical activity” and Maatoug et al. (82) used “twice-a-week physical activity sessions in school.”

#### 3.3.4. Material

Anthropometric measurements, in these mixed studies, systematically considered BMI for age and sex (e.g., BMI *z*-score, BMI percentile). Nevertheless, it was not the only measurement, because in three studies, this was combined with at least one other measurement of body composition [WC (three studies), skinfold thickness (two studies), impedance (one study)]. For example, Kokkvoll et al. (85) used “bioelectrical impedance,” in Morano et al. (83), “Skinfold thickness was determined (…) with a skinfold caliper,” and in Rito et al. (81), “waist circumference was obtained for every child.” With regard to PA, three studies clearly used an objective measurement coupled with a second self-reported one. This helped avoid over/underestimating the results. Sanders et al. (84) mentioned that “pre- and post-intervention fitness tests were administered to participants (…) program participants and their parents completed a physical activity and nutrition behavior questionnaire.” The other studies were based only on self-reported data [Maatoug et al. (82) mentioned that participants “responded to a 24 h food and physical activity recall questionnaire”] or no PA measurement was done in them (85). Three tools were mainly used to measure nutrition: questionnaire (two studies), 24 h recall (two studies), and a 7-day dietary diary (one study). For example, Morano et al. (83) reported, “dietary habits were assessed with a 7-day food diary.” Only in one study, nutritional measurement (85) was not performed.

#### 3.3.5. Efficacy

As mentioned previously, all mixed studies focused on secondary or tertiary prevention of obesity. One of the major objectives was therefore to influence downward BMI and weight in order to reduce the fat mass of the participants. Of the six programs, five had direct effects on BMI (81–85). One article did not show significant BMI reduction, even though it indicated a tendency to slow its growth. Rieder et al. (86) mentioned that percentile BMI measurements taken before and after the intervention indicated a general upward trend (*p* = 0.0003). Nevertheless, during the intervention period, the slope of the BMI percentile showed a downward trend (*p* = −0.0001). Moreover, a comparison of the results of the preintervention phase and the intervention phase showed significant variations (*p* = 0.003). “For intervals T12 to T0 vs. T0 to T9, there were significant decreases in rates of gain in BMI (0.13 vs. 0.04, *p* < 0.01, BMI percentile [0.0002 vs. −0.0001, *p* < 0.01].”

Each of these studies also presented at least one positive variation on one of the various health, physical, psychological, or nutritional factors. For example, Maatoug et al. (82) showed positive effects on PA, *p*-value (pre-post) = 0.001, and reduction of caloric intake; *p*-value (pre-post) < 0.001; Rito et al. (81) mentioned “vigorous physical activity (day/week), CI 95% 0.1–0.5, *p*-value = 0.008” and Morano et al. (83) showed that “Actual (*p* < 0.001) and perceived (*p* < 0.03) physical abilities, physical activity enjoyment (*p* = 0.03), and psychosocial HRQoL (*p* < 0.05) also improved from pre- to post-intervention.”

## 4. Discussion

### 4.1. Summary of the findings

This study identified a number of differences between the studies of the various IS settings (Table 4); these differences could be grouped into five elements (intervention team, prevention objective, duration, material, and results).

**Table 4:** Results summary.

	School/Community	Clinical	Mixed
Intervention team	Rarely multidisciplinary	Widely multidisciplinary	Totally multidisciplinary
Prevention goal	Primary/secondary (preventive)	Secondary/tertiary (curative)	Secondary/tertiary (curative)
Duration	Variable (53% more than a year; 39% less than 6 months)	Short (84% less than a year)	Relatively short (66% less than a year)
Material	Reliable	Reliable ++	Reliable +
Efficacy	Anthropometric measurements (72%) and health factors (90%)	Anthropometric measurements (74%) and health factors (83%)	Anthropometric measurements (83%) and health factors (100%)

Intervention team: The intervention team in the school/community was mostly composed of a single stakeholder that was often the teacher (54.5% of cases). In the clinical and mixed sectors, the ISs largely depended on a multidisciplinary team with various members specialized in health. In both sectors, stakeholders contributed to the success of the ISs as key actors.

Prevention objective: The school/community ISs mainly targeted primary prevention because there was no selection of participants, while participants in the other sector were chosen by targeted criteria such as overweight or obese (according to their BMI for age and sex), and the same was the case for mixed studies.

Duration: One interesting point in the school/community setting was that it allowed for a relatively long intervention duration, and ISs aimed at preventing obesity needed time to embed and develop before being evaluated (60). On the other hand, clinical and mixed ISs tended to last for a shorter period of time. More than three-fourths of the studies done in the clinical setting and two-third in the mixed setting lasted less than a year.

### 4.2. Findings: What can be understood and learned?

#### 4.2.1. Material

Clinical and mixed ISs tended to use more objective instruments, requiring more skills and knowledge. This allowed them to associate and combine certain measurements to achieve more accurate results and not over/underestimate their results.

#### 4.2.2. Efficacy

Many authors agreed (23–25) that school was a privileged place for prevention. Our results seem to confirm this tendency, because school/community ISs showed significant and promising results both on anthropometric measurements relative to obesity (72%) and on health-related factors (90%). The clinical setting was also a beneficial location for the treatment of obesity (secondary/tertiary preventions). This setting was seen as a source of quality and reliable information (17) and it provided important results on both obesity (74% of the studies) and health-related factors (83% of the studies) (87).

### 4.3. Prospects

In light of these findings, it is necessary that mixed studies should be prioritized, with a combination of school/community-based and clinical-based strengths. Indeed, our study found that mixed ISs provided the most promising results; 83% of the studies showed a positive influence on obesity and 100% on health-related factors. Nevertheless, those we considered sought to apply a relatively clinical model to the school/community setting (82) but did not participate in an exchange relationship, and therefore, the strengths of school/community-based ISs were “left behind.” To enhance global obesity prevention and in line with health recommendations to prevent childhood obesity, Table 5) proposes recommendations for future studies to be more effective.

**Table 5:** Recommendations for future ISs.

	Recommendations	Ideas for optimization	
		Clinical	School/Community
Intervention team	Multidisciplinary/specialized - Each contributor is specialized in their field or has the skills/knowledge to intervene (e.g., training) - ISs must be multidisciplinary and intervene on essential fields (i.e., PA, Nutrition, Psychology)	- Doctor - PA specialist - Psychologist - Dietician	- PE teacher - Other teachers - School nurses
Prevention goal	Primary/secondary/tertiary (global prevention) - ISs should focus on the entire youth population with the opportunity to identify young people at risk (material and skills/knowledge) in order to intervene and/or guide as well as possible - Possibility of further control examination not requiring the personal initiative of youth (examination by medical staff with more objective measuring equipment, to confirm or deny the presence of overweight or obesity)	Individual ISs Secondary prevention: - Additional individual consultation with a dietician every 3 months Tertiary prevention: - Start a clinical weight loss management program (after an “ability to change” assessment)	Collective ISs Primary prevention: - Control consultation with the school nurse every year - Availability of free water - Availability of fruits and vegetables - Posters - Flyers Secondary prevention: - Courses on obesity - Workshops on “how to eat healthy” with a dietician - Additional PA classes (new activities or tailored PA)
Duration	- Various - “Daily” integrated primary prevention - “Extended” primary/secondary prevention (1 year) renewable - “Shorter” tertiary prevention (<6 months) after needs assessment and with a follow-up. In addition to the two other axes mentioned above	Shorter interventions duration focusing on: - Life habits (enjoyable PA, gradual increase in difficulty, reduced sedentary time) - Nutrition (tailored diet to avoid the feeling of frustration, deprivation) - Psychology (climate free from judgment; behavioral therapy) At least a 3-month follow-up (Canadian recommendations)	Extensive duration focusing on: - Information - Environment
Material	- “Classic” measurement, estimated Measures in first report + possibility of further control if deemed necessary, with objective measurement	Impedance DXA Blood samples Interview Monitored stress test	BMI BMI for age and sex WtHR WC Questionnaires and field tests

ISs, intervention strategies; PA, physical activity PE, physical education; WC, waist circumference.

Clinical:

- Multidisciplinary team with specialists;
- Objective measurements;
- Relative efficiency to treat.

School/Community:

- Pleasure/enjoyment;
- Various activities;
- Relative efficiency to prevent;
- Time (place where children spend most of their time).

**Figure 2:**
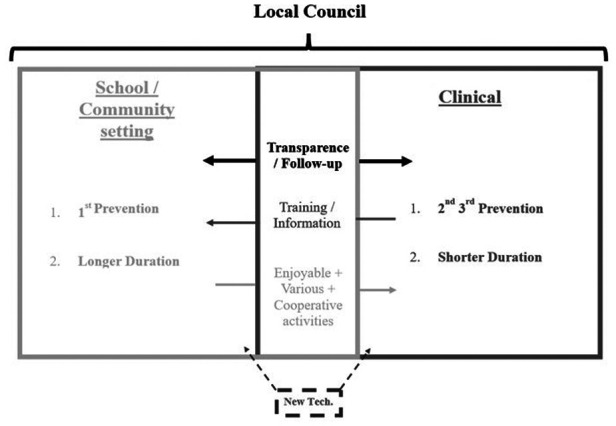
Modelling of physical activity ISs.

For the purposes of synthesis (Figure 2), we recommend the implementation of a transparent local council involving the entire local community (e.g., school children, representatives, clinical specialists, stakeholders, parents, associative representatives) (25) responsible for the development, improvement, and implementation of prevention programs at the local level. The most important point here is that each intervention sector should have its own prerogatives. Nevertheless, to achieve effective obesity prevention, the different settings need to function in a more transparent manner without ignoring the three different aspects of prevention (primary secondary = school/community; secondary tertiary = clinical). Another interesting aspect is the use of new technologies in prevention. On this point, further studies are needed to evaluate the potential added value of technological tools in obesity prevention.

### 4.4. Limitations

Many studies use BMI as a measurement variable or other variables related to it. However, although it is easy to use, BMI alone is not a representative indicator of the benefits of a program. Morano et al. (83) showed that the use of multiple body composition measurements provide better indications of changes in body fat, which is more representative of expected changes. Furthermore, weight-related measurements can skew the results (72). In PA-oriented programs, which lead to positive body composition change (e.g., lean mass gain and body fat loss), the participant weight can increase (lean mass is heavier), which can induce an increase in BMI measurements. This lack of precision relative to these measurements can also distort the results of the studies that use only BMI as the measurement variable or variables relative to the weight of the participants (22).

A second limitation could be the lack of information about programs in some studies to assess the quality of the study. This sometimes makes it difficult to identify the equipment used, the staff involved, and their skills/knowledge. The duration of the program is another limitation. Indeed, all the identified studies do not last the same amount of time (e.g., more than 1 year; less than 6 months; a few weeks). Furthermore, they do not use the same evaluation time (e.g., pre-post; pre-post + follow-up). It is, therefore, difficult to evaluate the effectiveness of one IS in relation to another over a short period of time. Moreover, to assess the efficacy of ISs, more time is required to embed them (60). The number of participants in each study is also highly variable and therefore can make a generalization or a comparison with other studies impossible. The last limitation pertains to the number of studies selected for each setting. Indeed, the number of studies being relatively low in the mixed setting can lead to an over/underestimation of the results. Nevertheless, this work seems to yield promising results, and future studies must continue to move to mixed setting, to nested ISs.

## 5. Conclusion

The main objective of this study was to propose a first combination and comparison of obesity prevention intervention programs from the clinical and the school or community sectors. However, our review showed that comparisons are difficult to make since the standards and units used for measurements are different and vary according to the protocols and areas of application. Nevertheless, we believe that this study offers an initial proposal for bridging the gap between the clinical and the school/community sectors, the two most promising sectors in terms of outcomes for obesity prevention in youth in particular.

Future studies should focus on establishing a prevention program in a given geographical area (e.g., town, county), involving all stakeholders with their respective skills/knowledge, in the decision-making process and in the development of ISs (e.g., parent association, professors, doctors, local representatives, sports association), so that it becomes the most efficient and best adapted to its environment. Although this study focused on physical activity interventions, it would be relevant to also look at nutrition interventions, since nutrition is a major theme in obesity prevention. The main objective of this study was to propose a first combination of obesity prevention intervention programs with the clinical and the school or community sectors. Our review showed that comparisons are difficult since the standards and units used to measure are different and vary according to the protocols and areas of application. However, we believe that this study offers an initial proposal for bridging the gap between the clinical and school/community sectors, the two most promising sectors in terms of outcomes for obesity prevention in youth in particular.

## Data Availability

The original contributions presented in the study are included in the article/Supplementary Material, further inquiries can be directed to the corresponding author.

## Appendix A Keywords and meshterms

**Table:**

Theme 1	Theme 2	Theme 3	Theme 4
Program	Obese	“Physical activity”	Child
Protocol	Obesity	Sport	Childhood
Plan	Overweight		Young
“Intervention strategy”	“Obesity Management”		Youth
Method			“Elementary school”
Recommendation			Children
Treatment			Adolescence
Prevention			Adolescent
			“under 18”
			Student
			Pediatric

## Appendix B Equations of research

**Table:**

((((program[Title/Abstract] OR “intervention strategy”[Title/Abstract] OR proto-col[Title/Abstract] OR method[Title/Abstract] OR plan[Title/Abstract] OR recommenda-tion[Title/Abstract] OR treatment[Title/Abstract] OR prevention[Title/Abstract])) AND (obese[Title/Abstract] OR obesity[Title/Abstract] OR overweight[Title/Abstract] OR “obesi-ty management”[Title/Abstract])] AND [“physical activity”[Title/Abstract] OR sport[Title/Abstract]]) AND (Child[Title/Abstract] OR childhood[Title/Abstract] OR young[Title/Abstract] OR youth[Title/Abstract] OR “elementary school”[Title/Abstract] OR children[Title/Abstract] OR adolescent[Title/Abstract] OR adolescence[Title/Abstract] OR under 18[Title/Abstract] OR student[Title/Abstract] OR pediatric[Title/Abstract]) AND ([Observational Study[ptyp] OR Multicenter Study[ptyp] OR Clinical Study[ptyp] OR Clinical Trial[ptyp] OR Comparative Study[ptyp] OR Evaluation Studies[ptyp] OR Gov-ernment Publications[ptyp] OR Letter[ptyp] OR Meta-Analysis[ptyp] OR Randomized Controlled Trial[ptyp] OR Validation Studies[ptyp]] AND full text[sb] AND “last 5 years”[PDat] AND Humans[Mesh] AND [English[lang] OR French[lang]] AND [child[MeSH:noexp] OR adolescent[MeSH]])
